# U.S. patient preferences for long‐acting HIV treatment: a discrete choice experiment

**DOI:** 10.1002/jia2.26099

**Published:** 2023-07-13

**Authors:** Susan M. Graham, Douglas Barthold, Brett Hauber, Aaron T. Brah, Enrique Saldarriaga, Ann C. Collier, Rodney J. Y. Ho, the Emory PREFER Team, Vincent C. Marconi, Jane M. Simoni

**Affiliations:** ^1^ Division of Allergy & Infectious Diseases Department of Medicine University of Washington Seattle Washington USA; ^2^ Department of Global Health University of Washington Seattle Washington USA; ^3^ Department of Epidemiology University of Washington Seattle Washington USA; ^4^ The Comparative Health Outcomes, Policy, and Economics (CHOICE) Institute University of Washington Seattle Washington USA; ^5^ Pfizer, Inc New York New York USA; ^6^ Department of Pharmaceutics and Bioengineering University of Washington Seattle Washington USA; ^7^ Division of Infectious Diseases Department of Medicine Emory University School of Medicine Atlanta Georgia USA; ^8^ Department of Global Health Rollins School of Public Health Emory University Atlanta Georgia USA; ^9^ Department of Psychology University of Washington Seattle Washington USA

**Keywords:** antiretroviral agents, antiretroviral therapy, choice behaviour, delayed‐action preparations, HIV, patient preference

## Abstract

**Introduction:**

Recent advances in long‐acting antiretroviral therapy (LA‐ART) could provide new options for HIV treatment and reduce adherence barriers, if regimens are acceptable to patients. We elicited preferences for key attributes of potential LA‐ART regimens among people with HIV (PWH) in the United States, focusing on four treatment modes (oral tablets, subcutaneous injections, intramuscular injections, and implants), product characteristics and location of administration.

**Methods:**

A discrete choice experiment was conducted among PWH aged ≥18 years recruited from HIV clinics in Washington State and Atlanta, Georgia from March 2021 to June 2022. Participants responded to 17 choice scenarios, each with three options: two systematically generated hypothetical LA‐ART regimens and a constant opt‐out (their current daily oral treatment). LA‐ART regimen descriptions included treatment mode, pain, dosing frequency, location, pre‐treatment time with undetectable viral load, pre‐treatment negative reaction testing and “late‐dose leeway” (i.e. flexibility or forgiveness in timing the next dose). We used conditional logistic regression, with an interaction between treatment mode and pain, to estimate preference weights for all attribute levels.

**Results:**

Seven hundred participants (350 at each site) enrolled, with median age 51 years (range 18–73); 70% identified as cisgender male, 24% as cisgender female and 6% as non‐binary or transgender. LA oral tablets were the only mode preferred over current daily oral treatment, with annual implants and injections the next most preferred LA‐ART option. Longer time between doses was preferred, and administration at home was preferred to clinics, which were preferred to pharmacies. Attributes with less impact on preferences included oral lead‐in treatment to achieve viral suppression or test for negative reactions and late‐dose leeway around the prescribed dosing interval. Participants in Atlanta were more likely to prefer their current daily oral ART than participants from Seattle.

**Conclusions:**

PWH in the United States may soon have several options for LA‐ART. Our results suggest that LA oral tablets will be preferred by many patients over their current daily oral treatment, while implants and injections with longer duration may be acceptable to some. Future research should investigate sources of preference heterogeneity and actual uptake of and adherence to LA‐ART products, when available.

## 1 INTRODUCTION

In 2020, the Centers for Disease Control and Prevention estimated that for every 100 people who were diagnosed with HIV, only 74 had received HIV care and 65 were virally suppressed, based on their most recent viral load result [1]. Disparities in U.S. HIV care cascade outcomes (i.e. HIV diagnosis, engagement in care and viral suppression) have been reported among adolescents and young adults [2, 3], Blacks [2, 3, 4, 5], Hispanic persons [6], injection drug users [3], women [3], heterosexual men [3, 4], foreign‐born persons [4] and those of low socio‐economic status [3]. New approaches to optimizing antiretroviral therapy (ART) uptake, adherence and retention are clearly needed, in order to overcome challenges that undermine treatment success, such as HIV stigma, treatment fatigue, missed visits or refills, forgetfulness and side effects [7, 8, 9].

Recent advances in long‐acting antiretroviral therapy (LA‐ART) could provide new options for HIV treatment and reduce adherence barriers, if regimens are acceptable to patients. Initial results from the first LA‐ART regimen to reach the market have been promising. In the LATTE‐2 randomized, open‐label phase 2b trial, participants were randomly assigned (2:2:1) to receive two intramuscular injections of long‐acting cabotegravir plus rilpivirine at 4‐ or 8‐week intervals or a comparable daily pill‐based regimen [10]. The regimen at either frequency was as effective as daily oral combination therapy at maintaining HIV viral suppression through 96 weeks [10]. This regimen was well accepted and tolerated, with 97% of 254 participants reporting 5 or 6 on a 6‐point scale of treatment satisfaction and 99% stating they would be “highly satisfied to continue” their long‐acting injectable (LAI) ART [10]. In a qualitative study associated with this trial, 39 in‐depth interviews were conducted with participants and providers from the United States and Spain [11]. Despite commonly experienced injection site reactions [10], participants were generally tolerant of the regimen, finding injections convenient, with reduced potential for HIV disclosure and elimination of the “daily reminder of living with HIV” [11].

Due to the early success of the cabotegravir/rilpivirine regimen, research on patient acceptance and preferences has focused on injectable regimens. For example, Williams and colleagues surveyed 400 adults taking daily ART at two clinical sites, reporting that 61%–85% would “definitely or probably” try LAI ART, depending on the dosing interval, with over one quarter saying they would try an injectable regimen even if it cost “much more” than their current regimen [12]. In a survey conducted by an Italian patient advocacy group [13], Rusconi et al. reported that 55% of the 488 respondents knew about LAI ART and 83% would appreciate not taking pills on a daily basis. Furthermore, 30% said they would benefit even if hospital‐based injections were required every month and an additional 39% would benefit if hospital‐based injections were required every 2 months [13]. While the injectable cabotegravir/rilpivirine regimen was first on the market, a number of promising LA‐ART products are in development that could result in effective combination regimens with less frequent administration, fewer injections or other potential advantages [14]. Our recent interviews with key informants involved in HIV drug development and clinical trials revealed that experts thought implants, subcutaneous injections and long‐acting oral tablets could also become available for HIV treatment in the near future [15]. Data on patient preferences for this wider range of LA‐ART modalities are currently sparse.

Discrete choice experiments (DCEs) are used to measure and quantify preferences in the absence of revealed preference data from field studies [16, 17]. In a DCE, individuals are asked to choose between different hypothetical alternatives, each defined by a set of attributes with varying levels. Responses can be used to determine whether participants’ preferences are significantly influenced by the attributes, the relative importance of each attribute and the trade‐offs (i.e. marginal rates of substitution) patients are willing to make among attributes [18]. DCEs may be particularly helpful when interventions are still in development or when a comparison of multiple different options in observational studies or trials would be too costly or impractical. Therefore, the results of a DCE conducted at an early stage of product development can generate important insights for product developers and help inform features that will increase acceptability.

To obtain patient preferences on a range of potential long‐acting treatment modalities without over‐emphasizing the first LA‐ART regimens on the market, we conducted key informant interviews to identify treatment modalities most likely to become available in the next 5–10 years [15]. Based on this qualitative work and our prior research on LAI ART acceptability with both patients and providers [19, 20], we developed and pilot‐tested a DCE to capture the preferences of patients engaged in HIV care with respect to key attributes of these long‐acting regimens [21]. The results of the pilot testing informed the experimental design of a fully deployed DCE at research sites in Seattle, Washington and Atlanta, Georgia. Our objective in this analysis is to present the results of this fully deployed DCE, which investigated preferences related to four different treatment modalities (oral tablets, subcutaneous injections, intramuscular injections and implants) and their characteristics, compared to patients’ current daily oral HIV regimen.

## 2 METHODS

### 2.1 Population and setting

We recruited DCE participants from the University of Washington HIV clinics in Bremerton, Everett, Federal Way, Olympia, and Seattle, Washington and from Emory University's Infectious Diseases Program in Atlanta, Georgia. Recruitment was conducted between March 2021 and June 2022 through outreach to patients by e‐mail or telephone using contact information from the HIV patient registries at each site, or in person at their regular clinic appointments. Our target enrolment was 350 at each site, for 700 participants overall. Eligibility criteria included living with HIV, age ≥18 years, established care at one of the research sites, fluency in English and ability to provide informed consent. Exclusion criteria included currently taking an LAI regimen and being an “elite controller” who has a very low viral load without requiring ART (approximately 0.1%–2.5% of all HIV infections worldwide [22]). In addition, individuals judged to be cognitively impaired or under the influence of drugs or alcohol during in‐person screening were excluded.

### 2.2 Ethical oversight

The University of Washington (UW) and Emory University reviewed and approved the study protocol and informed consent documents, with the UW serving as the single institutional review board of record (STUDY00007390). All participants provided electronic informed consent.

### 2.3 DCE design

We developed our DCE based on feedback from key informants about potential treatment modes and their likely frequency of administration [15]. This feedback led to the selection of four LA‐ART treatment modes (long‐acting oral tablets, subcutaneous injections, intramuscular injections and implants) and six additional attributes: dosing frequency, location of treatment administration, pain with administration/insertion, pre‐treatment time undetectable (should viral suppression be required before initiating LA‐ART), pre‐treatment “negative reaction” testing (implementing an oral lead‐in to assess tolerability or to exclude reactions such as “an allergic rash or abnormal liver test results” should this be required before starting LA‐ART) and “late‐dose leeway” (i.e. flexibility or forgiveness in dosing timing before breakthrough viremia). These concepts were carefully explained to DCE participants in the survey introduction (details in Supporting Information 1).

Attribute levels were restricted based on what key informants considered feasible. For long‐acting oral medications, attributes were restricted to: no pain, administration at home and frequency 1 or 4 weeks. For subcutaneous injections, attributes were restricted to: no or mild pain; administration at home, clinic or pharmacy; and frequency 1, 4, 8 or 12 weeks. For intramuscular injections, attributes were restricted to: mild or moderate pain; administration at clinic or pharmacy; and frequency 4, 8 or 12 weeks. For implants, attributes were restricted to: mild or moderate pain, insertion at clinic and frequency 26 or 52 weeks. If the location was clinic or pharmacy, the dosing frequency was restricted to ≥4 weeks. The other attributes had no restrictions. Choices for pre‐treatment time undetectable were 0, 3 and 6 months. Negative reaction testing was needed or not needed. Late‐dose leeway was a duration of time set at 50% or 100% of the dosing interval for that specific LA‐ART option, with 50% referred to as a “short” late‐dose leeway and 100% referred to as a “long” late‐dose leeway.

### 2.4 Survey components

The DCE survey (Supporting Information 1) was pilot tested with 50 participants over a series of waves with iterative improvements [21]. The survey started with an overall introduction, then introduced each treatment mode or “option,” along with visual images. The ability to remove an implant if a negative reaction occurred was included in the description of that treatment mode. Because effective HIV treatment requires two or more antiretroviral medications and to avoid mixing treatment modes, we advised participants that each hypothetical LA‐ART regimen would require two products, both administered by the same mode [15]. This introduction was followed by three comprehension questions about these treatment options to ensure understanding. Next, the first three attributes or “features,” including location of treatment, frequency of dosing and pain experienced, were introduced. Participants were also asked to make the following assumptions as they considered their choices: (1) all options would work equally well (i.e. could suppress viral load but not cure HIV); (2) there would be no difference in costs compared to participants’ current regimen; and (3) the safety of treatment administration locations would not be impacted by COVID‐19. An instructional video with narrative descriptions explaining how the choice sets were to be read presented a practice choice set with only the treatment modes and the first three attributes. After this practice choice set, pre‐treatment time undetectable and pre‐treatment negative reaction testing were introduced, followed by a comprehension question, then finally late‐dose leeway was introduced, followed by its own comprehension question. Another instructional video introduced the more complicated choice sets with all these attributes, followed by another practice choice set.

**Figure 1:**
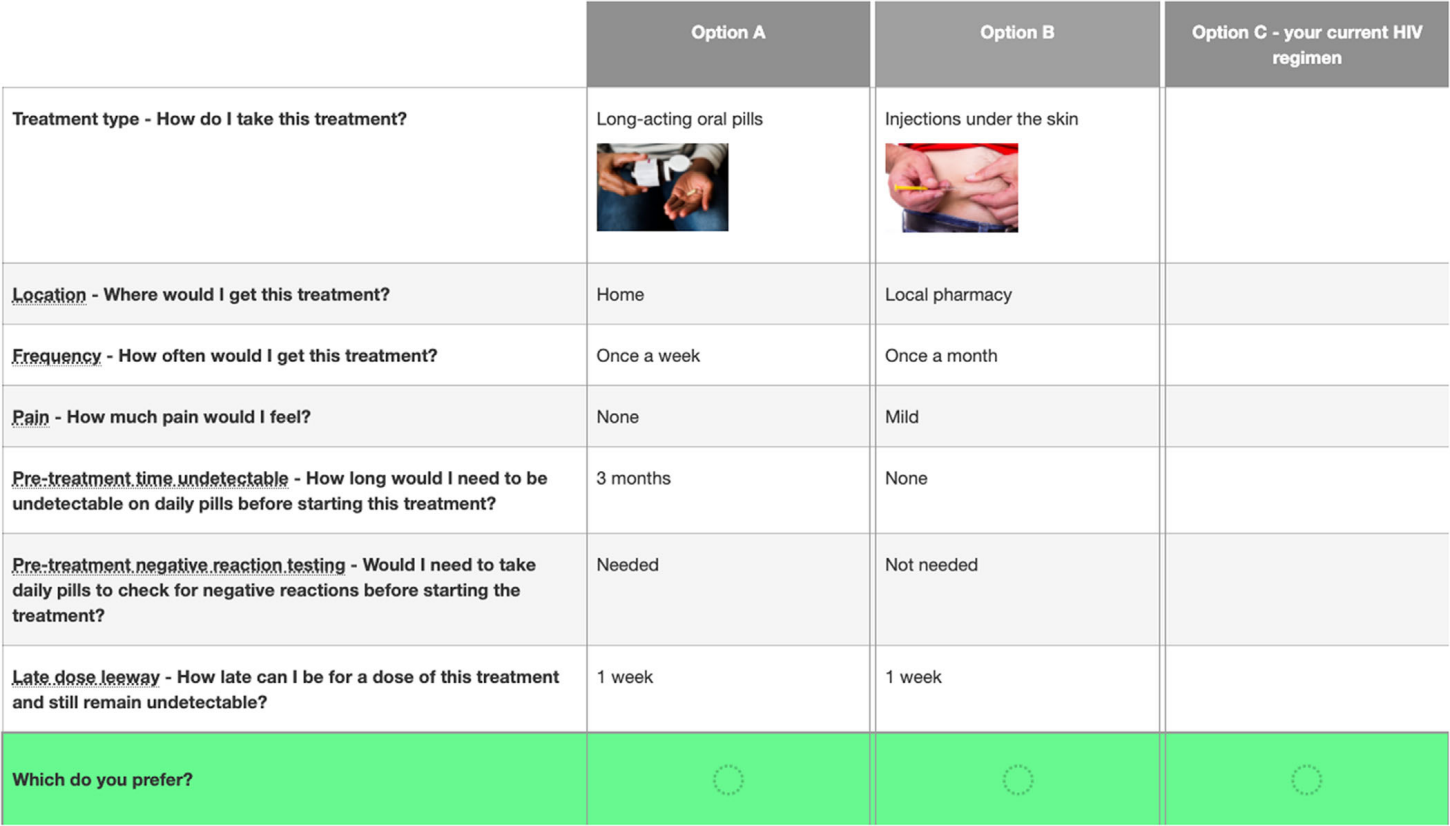
Example choice set presenting two different long‐acting antiretroviral therapy (LA‐ART) regimens (Option A and Option B) and the constant Option C opt‐out (current daily oral regimen). For each LA‐ART option, attributes presented included treatment type, location, frequency, pain, pre‐treatment time undetectable, pre‐treatment negative reaction testing and late‐dose leeway. “Long” late‐dose leeway was defined as 100% of the dosing interval and “short” late‐dose leeway was defined as 50% of the dosing interval for that specific treatment option.

DCE participants responded to 17 choice scenarios, each with three options: two systematically generated hypothetical LA‐ART regimens and a constant opt‐out (i.e. the participant's current daily oral treatment). Figure 1 presents an example of the choice sets used. Participants were randomized to 1 of 4 blocks of 16 choice scenarios (out of 64 possible), which were presented in a random order. The 17th question presented two different types of long‐acting oral regimens that were compared to the same constant opt‐out. The DCE was designed using Ngene software (ChoiceMetrics, Sydney, Australia). The experimental design was unlabelled, and was constructed using a modified Federov algorithm and D‐optimal main effects [21].

### 2.5 Data collection

Individuals could access the survey at home through an e‐mailed invitation link or in a private area within the clinic (after COVID‐19 restrictions on in‐person research were lifted). Research staff were available for assistance if needed, either by telephone or in‐person (for clinic participation). Participants were screened for eligibility and consented, if eligible, using a questionnaire available in REDCap, an electronic data capture tool hosted by the UW. After consenting, participants were linked from REDCap directly to the DCE survey, which was administered in SurveyEngine (SurveyEngine GmbH, Berlin, Germany), an online data collection platform specifically designed for preference research. After the introduction described above and before being presented with the 17 choice scenarios, participants were asked questions about their HIV history, their current and past ART regimens, and their experience with injections, pill storage and clinic visits. At the end of the choice sets, additional data were collected on quality of life, provider support, social support, socio‐demographic characteristics and preferences for reminders about clinic visits or treatment administration. In addition, six questions on internalized stigma were included, based on a validated scale [23]. If the participant consented to chart linkage, clinical data, including the participant's current HIV regimen, most recent CD4 count, most recent viral load and number of HIV and non‐HIV medications taken daily, were also abstracted from the medical record.

### 2.6 Data analysis

Descriptive statistics were used to summarize participant characteristics, overall and by site, with comparisons across sites using independent‐sample *t* tests for continuous variables and Chi‐square or Fisher exact tests for categorical variables. Each HIV stigma question was scored from 1 (no stigma) to 4 (high‐level stigma), and an average score for all stigma questions answered was calculated. Conditional logistic regression was used to analyse the participants’ choices across all tasks using attribute levels as the covariates. Data were clustered by participant, to account for intra‐individual correlation. All attribute levels were categorical effects‐coded, with the omitted level estimated from the negative sum of all other levels in the model. The primary endpoints were all attribute‐level preference weights, with 95% confidence intervals (CI). Log likelihood ratio tests and Akaike's Information Criterion were used to assess model fit [24]. Due to restrictions on the pain attribute by treatment mode, we included an interaction between treatment mode and pain. The best fitting model coded injection mode as a single type (combining subcutaneous and intramuscular) while restricting implants to 6‐ or 12‐month frequency. We conducted similar analyses stratified by site, in order to explore differences between preference weights for the participants in Georgia and those in Washington State. Mean preference‐weight estimates relative to the mean attribute effect normalized around zero and with 95% CI were graphed for each attribute. All data analyses were performed using Stata version 17.0 (StataCorp LLC, College Station, TX) or R version 4.2.2 (https://www.r‐project.org/about.html).

## 3 RESULTS

Seven hundred participants enrolled, with 350 at each site. Table 1 presents the characteristics of this population, overall and by site. The median age was 51 years, ranging from 18 to 73. Overall, 70% of participants identified as cisgender male, 24% as cisgender female and 6% as non‐binary, transgender or other. There were large differences by site, with Atlanta participants less likely to be of Hispanic ethnicity and more likely to be Black, female, heterosexual, unemployed or uninsured. Participants in Atlanta had had HIV and been on ART for a longer duration, had lower recent CD4 counts, were more likely to report having been diagnosed with AIDS and were less likely to have undetectable viral load, despite being more likely to take only one HIV pill per day. In addition, Atlanta participants were more likely to choose their current daily oral regimen over the LA‐ART options presented and were more likely to take the DCE survey in the clinic with assistance from research staff.

**Table 1:** Characteristics of 700 participants by study site

	Total (*N* = 700)	Atlanta (*n* = 350)	Seattle (*n* = 350)	*p* Value for comparison
Age (years)				
Mean (SD)	48.6 (12.1)	49.3 (12.2)	48.0 (12.0)	0.15
Median [min, max]	51 [18, 73]	51 [18, 72]	50 [22, 73]	
Hispanic ethnicity, *N* (%)				
No	623 (89.0%)	325 (92.9%)	298 (85.1%	0.001
Yes	61 (8.7%)	17 (4.9%)	44 (12.6%)	
Missing	16 (2.3%)	8 (2.3%)	8 (2.3%)	
Race, *N* (%)				
White	259 (37.0%)	23 (6.6%)	236 (67.4%)	<0.001
Black	331 (47.3%)	295 (84.3%)	36 (10.3%)	
Other/Mixed	94 (13.4%)	24 (6.9%)	70 (20.0%)	
Prefer not to say	16 (2.3%)	8 (2.3%)	8 (2.3%)	
Gender, *N* (%)				<0.001
Woman	168 (24.0%)	135 (38.6%)	33 (9.4%)	
Man	493 (70.4%)	197 (56.3%)	296 (84.6%)	
Transgender woman	15 (2.1%)	8 (2.3%)	7 (2.0%)	
Transgender man	6 (0.9%)	4 (1.1%)	2 (0.6%)	
Other	10 (1.4%)	3 (0.9%)	7 (2.0%)	
Prefer not to say	8 (1.1%)	3 (0.9%)	5 (1.4%)	
Sexual orientation, *N* (%)				<0.001
Heterosexual	227 (32.4%)	173 (49.4%)	54 (15.4%)	
Lesbian, gay or bisexual	419 (59.9%)	143 (40.9%)	276 (78.9%)	
Prefer not to say	30 (4.3%)	24 (6.9%)	6 (1.7%)	
Missing	24 (3.4%)	10 (2.9%)	14 (4.0%)	
Employment, *N* (%)				<0.001
Full time	192 (27.4%)	55 (15.7%)	137 (39.1%)	
Part time	79 (11.3%)	33 (9.4%)	46 (13.1%)	
Not working	407 (58.1%)	248 (71.0%)	159 (45.4%)	
Prefer not to say	22 (3.1%)	14 (4.0%)	8 (2.3%)	
Any health insurance, *N* (%)	577 (82.4%)	237 (67.7%)	340 (97.1%)	<0.001
HIV stigma score	2.17 (0.79)	2.14 (0.80)	2.20 (0.77)	0.33
Time on ART (years)				0.035
Mean (SD)	15.6 (8.7)	16.3 (8.9)	14.9 (8.5)	
Missing	10 (1.4%)	3 (0.9%)	7 (2.0%)	
Time with HIV (years)				0.026
Mean (SD)	17.9 (9.8)	18.7 (9.9)	17.0 (9.6)	
Most recent CD4 count (cells/mm^3^)				0.0006
Mean (SD)	601 (316)	558 (338)	641 (289)	
Missing	30 (4.3%)	25 (7.1%)	5 (1.4%)	
Self‐reported AIDS diagnosis, *N* (%)^a^	292 (41.7%)	164 (46.9%)	128 (36.6%)	0.0006
Viral load undetectable, *N* (%)	500 (71.4%)	224 (64.0%)	276 (78.9%)	<0.001
HIV pills per day, *N* (%)				<0.001
One tablet	390 (55.7%)	230 (65.7%)	160 (45.7%)	
Two tablets	200 (28.6%)	72 (20.6%)	128 (36.6%)	
Three or more tablets	92 (13.1%)	39 (11.1%)	53 (15.1%)	
Prefer not to say/missing	9 (2.6%)	9 (2.6%)	18 (9.2%)	
Number of scenarios in which Option C (the constant opt‐out) was chosen, Mean (SD)	5.4 (6.4)	6.3 (7.0)	4.6 (5.7)	0.0004
Respondents selecting Option C (the constant opt‐out) at least once, *N* (%)	381 (54.4%)	181 (51.7%)	200 (57.1%)	0.149
DCE participation site, *N* (%)				<0.001
Home	299 (42.7%)	34 (9.7)	265 (75.7)	
Clinic	376 (53.7%)	312 (89.1)	64 (18.3)	
Other	25 (3.6%)	4 (1.1)	21 (6.0)	

Note: Comparisons were made across sites using independent‐sample *t* tests for continuous variables and Chi‐square or Fisher exact tests for categorical variables. SD: standard deviation; DCE: discrete choice experiment.

^a^ Data were missing for 14 participants, 9 from Atlanta and 5 from Seattle.

**Figure 2:**
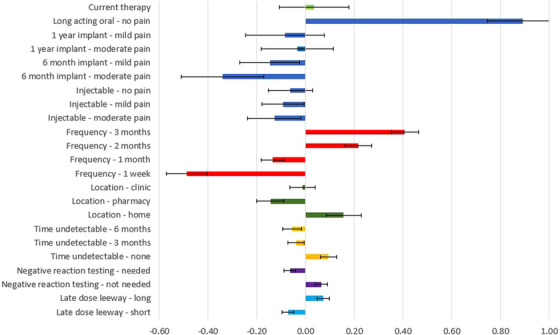
Long‐acting antiretroviral treatment (LA‐ART) preference weights from conditional logistic regression, entire study population. Mean preference‐weight estimates for each attribute relative to the mean attribute effect are presented, normalized around zero. Black lines with bars indicate 95% confidence intervals for preference weights. Positive weights indicate higher preference relative to the other levels evaluated. The overall relative importance of an attribute overall is the difference between the largest and the smallest preference weights of that attribute. “Long” late‐dose leeway was defined as 100% of the dosing interval and “short” late‐dose leeway was defined as 50% of the dosing interval for that specific treatment option.

Table 2 presents the mean preference weights for each attribute and level in the entire study population, along with 95% CI that allow comparison of utility across attribute levels. These results are also summarized in Figure 2. Across all participants, LA oral tablets were the only mode strongly preferred over current daily oral treatment, with a preference weight of 0.89 (95% CI 0.75, 1.04) for LA oral tablets, compared to 0.03 (95% CI −0.11, 0.18) for current treatment. Annual implants (preference weights −0.08 [95% CI −0.25, 0.08] and −0.03 [95% CI −0.18, 0.11]) for those with mild and moderate pain, respectively) and injections with no pain (preference weight −0.06 [95% CI −0.15, 0.03]) were next most preferred, with 95% CI for each overlapping current daily oral treatment. Longer time between doses was preferred, with clear separation between the 95% CI for 3 monthly, 2 monthly, monthly, and weekly, but overlap between 95% CI for 6‐ and 12‐month implants regardless of the pain. Administration at home (preference weight 0.16 [95% CI 0.08, 0.23]) was preferred to clinics (preference weight −0.01 [95% CI −0.06, 0.04]), which were preferred to pharmacies (preference weight −0.14 [95% CI −0.20, −0.09]), again with separation between the 95% CI for these three attribute levels. Attributes that impacted preferences less included oral lead‐in treatment to achieve viral suppression or test for negative reactions and late‐dose leeway around the prescribed dosing interval, although in each case, there was a clear preference (for no pre‐treatment requirements and for longer late‐dose leeway), with non‐overlapping 95% CI.

**Table 2:** Point estimates and 95% confidence intervals (CI) from conditional logistic regression for preference weights, entire study population

	Preference weight	95% CI
Current therapy (alternative‐specific constant)	0.03	−0.11, 0.18
Long‐acting oral—no pain*	0.89	0.75, 1.04
1‐year implant —mild pain	−0.08	−0.25, 0.08
1‐year implant—moderate pain	−0.03	−0.18, 0.11
6‐month implant—mild pain	−0.15	−0.27, −0.02
6‐month implant—moderate pain	−0.34	−0.51, −0.17
Injectable—no pain	−0.06	−0.15, 0.03
Injectable—mild pain	−0.09	−0.18, −0.01
Injectable—moderate pain	−0.13	−0.24, −0.02
Frequency—3 months	0.41	0.35, 0.46
Frequency—2 months	0.22	0.16, 0.27
Frequency—1 month	−0.14	−0.18, −0.09
Frequency—1 week*	−0.49	−0.57, −0.40
Location—clinic	−0.01	−0.06, 0.04
Location—pharmacy	−0.14	−0.20, −0.09
Location—home*	0.16	0.08, 0.23
Time undetectable—6 months	−0.05	−0.09, −0.02
Time undetectable—3 months	−0.04	−0.07, −0.01
Time undetectable—none*	0.09	0.06, 0.13
Negative reaction testing—needed	−0.06	−0.09, −0.04
Negative reaction testing—not needed*	0.06	0.04, 0.09
Late‐dose leeway—long^a^	0.07	0.05, 0.10
Late‐dose leeway—short*, ^a^	−0.07	−0.10, −0.05

Notes: Thin lines separate the different attributes assessed. Preference weights are relative to the mean effect. Non‐overlapping 95% confidence intervals for preference weights of different levels of the same attribute indicate significantly different utilities between the two levels compared.

^a^ Late‐dose leeway was defined as the flexibility or “forgiveness” in dosing timing before breakthrough viremia, with long leeway defined as 100% of the dosing interval and “short” leeway defined as 50% of the dosing interval for that specific treatment option.

* For each attribute, the omitted level, which was estimated from the negative sum of all other levels in the model, is indicated by an asterisk.

In general, Seattle participants had stronger preferences for LA‐ART regimens than Atlanta participants (Tables S1 and S2, and Figure S1). Most strikingly, while Seattle participants were somewhat negative about their current regimens (preference weight −0.26 [95% CI −0.46, −0.06]), Atlanta participants were more positive (preference weight 0.32 [95% CI 0.12, 0.52]). There was a clear preference for long‐acting oral treatment among Seattle participants, while implants and injectable regimens had 95% CI overlapping that of current therapy, indicating similar utility. The four levels of dosing frequency each had clear separation from the others at this site, with a stronger preference for each increase in the dosing interval. Also among Seattle participants, there was a clear preference for treatment at home, but the 95% CI for pharmacy and clinic overlapped, indicating similar utility for each location. In Atlanta, the 95% CI for long‐acting oral treatment overlapped that of current therapy, and implants were less preferred, with a 95% CI less than that of current therapy for all four implant options. The 95% CI for all injection options overlapped with that of current therapy, indicating similar utility. While longer dosing intervals were preferred in Atlanta, there was an overlap between 95% CI for 2 and 3 months and between 95% CI for 1 week and 1 month. There was a clear preference for clinic and home over pharmacy, but the 95% for clinic and home overlapped for Atlanta participants. Finally, although longer late‐dose leeway was clearly preferred in Atlanta, participants at this site had no clear preference in terms of pre‐treatment time undetectable and pre‐treatment negative reaction testing.

## 4 DISCUSSION

In this diverse population of 700 people with HIV (PWH) engaged in care at our study sites, we found that participants strongly preferred long‐acting preparations with longer intervals between dosing, less pain with administration, and greater privacy and convenience in terms of administration. In addition, participants preferred minimal barriers to LA‐ART initiation (i.e. no lead‐in testing for reactions or a requirement for viral load suppression) and greater tolerance for delays in product administration. There were differences in preferences across sites, with participants in Seattle more open to switching from their current daily oral regimen than in Atlanta. However, preferences for the different treatment modes when presented with the restrictions used in this DCE were fairly consistent, with more participants preferring long‐acting oral tablets than injections, and more participants preferring injections than implants.

To our knowledge, this is the first DCE investigating the preferences of PWH in the United States with respect to a broad range of potential LA‐ART regimens, including long‐acting oral tablets, subcutaneous and intramuscular injections, and implants. One published study evaluated the acceptability of LA‐ART in general, and asked participants to choose the one mode they would most prefer, “if all options cost the same and worked equally well” [25]. The options provided were oral pills, injections given every 1–2 months, implants inserted every 6–12 months or an intravenous infusion administered every 2–4 weeks. Among 374 participants, 61% reported that they were likely or very likely to use LA‐ART; 41% preferred pills, 40% preferred injections and 18% preferred an implant, with only 1% preferring an infusion [25]. While these results are interesting, the method used did not include trade‐offs between different products with varying attributes, such as frequency and site of administration, so results should be interpreted with caution. Our study extends that work and adds to the literature by using a rigorous DCE methodology to elicit patient preferences.

While long‐acting oral tablets were most preferred overall in our study, it is unclear when a long‐acting oral HIV treatment regimen will become available. Gilead Sciences and Merck have developed an LA‐ART regimen composed of weekly oral lenacapavir combined with weekly oral islatravir; a randomized trial (NCT05052996) of this regimen among PWH with viral suppression at baseline is ongoing, with a lower dose of islatravir than initially planned (due to toxicity) and an estimated completion date of December 2027 [26]. Currently, there is only one complete LA‐ART regimen on the market: the cabotegravir/rilpivirine regimen (Cabenuva, Viiv Healthcare, Brentford, United Kingdom). This regimen was approved by the U.S. Food and Drug Administration in 2021 with a once‐monthly dosing schedule [27], based on the ATLAS and FLAIR randomized controlled trials demonstrating the equivalence of this regimen to standard daily oral ART [28, 29]. The FDA approved every 2‐month administration of injectable cabotegravir/rilpivirine in 2022 [30], based on results of the ATLAS‐2 M study [31], which demonstrated the non‐inferiority of this dosing schedule. Viiv Healthcare is working on drug delivery technology to enable the delivery of larger doses with less pain, leading to an “ultra‐long‐acting” product dosed every 3 months or longer [32]. Evidence suggests that these regimens are tolerable in practice: in the week 124 FLAIR study, while injection site reactions were the most common adverse event (occurring after 21.3% of injections across all participant visits), they were generally mild (grade 1 or 2), short‐lived (median duration 3 days) and less frequent over time, and only 2.1% (11 of all 515 participants) discontinued LA cabotegravir/rilpivirine due to injection site pain or reactions [33]. While less frequent dosing and only mild pain could overcome concerns about injections, LA oral regimens have other advantages, such as greater convenience and privacy with home administration, that will likely influence preferences.

Participants in our DCE preferred LA‐ART regimens without a requirement for viral suppression before initiation, although this attribute had a relatively weak influence on choices. While each potential LA‐ART regimen we examined could lead to increased patient satisfaction if available, the extent to which LA‐ART regimens will address gaps in ART adherence and help attain viral suppression targets remains unclear [34]. Of note, in the study by Dandachi et al. asking participants to select their most preferred LA‐ART type, the likelihood of LA‐ART use did not correlate with adherence or viral suppression [25]. An ongoing AIDS Clinical Trial Group study called “LATITUDE” (NCT03635788) is investigating the use of injectable cabotegravir/rilpivirine compared to daily oral therapy among patients with a history of poor adherence, who were specifically excluded from prior trials [35]. While LATITUDE was originally designed with an oral lead‐in period of 6 months during which conditional economic incentives would promote viral suppression [35], new evidence suggests that viral suppression may not always be needed prior to starting cabotegravir/rilpivirine [36]. Results of the LATITUDE trial and other studies could provide important evidence on the extent to which LA injectable treatment may address current gaps in viral suppression. Further research on preferences for LA‐ART that includes populations who have struggled with adherence and viral suppression, such as adolescents and those with substance use or mental health problems, will be important as new regimens become available.

Our DCE participants also preferred LA‐ART regimens without a requirement for allergic reaction or other side effect testing before initiation and those with some “leeway” or forgiveness in dosing. Of note, the requirement for oral lead‐in for “negative reaction” testing has been waived for injectable cabotegravir/rilpivirine, based on the week 124 FLAIR results [33]. While an oral lead‐in period to identify and manage potential adverse drug reactions is no longer an issue for cabotegravir/rilpivirine, it may well be needed for other regimens. In addition, missed or late administration could lead to antiretroviral resistance mutations, regardless of treatment mode [34]. Resistance to injectable cabotegravir/rilpivirine has been reported for a small number of participants in recent clinical trials [37]. While they may be less influential for patients, concerns about managing adverse reactions and ensuring adequate leeway in the event of a missed dose will be key attributes influencing the preferences of providers.

Switching to LA‐ART may not be of interest to patients who are doing well on their current daily regimen. Indeed, in a separate analysis exploring associations with selecting the “Option C” constant opt‐out in this DCE, we found that PWH who were older, more adherent, more averse to injections and had lower educational attainment more frequently chose their current daily regimen in this DCE [38]. A striking finding of the current study is the difference in preferences by geographic site, which were evaluated nominally in stratified analysis for this manuscript, with no direct statistical comparison. Atlanta participants were more positive about their current daily oral regimen than were Seattle participants, and their preferences for treatment mode were less strong, with the 95% CI for current regimen overlapping those of long‐acting oral treatment and injections. While they clearly did not prefer implants, they also did not have a preference for home over the clinic. Atlanta participants were more likely to be unemployed and uninsured, and gaps in insurance coverage have led to challenges implementing injectable cabotegravir/rilpivirine at the Atlanta site [39]. These structural problems may underlie patients’ stronger preference for their current daily oral therapy, despite our request that patients assume there would be no difference in cost for the LA‐ART options presented relative to their current daily oral regimen. Site differences may also have been influenced by the greater proportion of cisgender women in Atlanta and their experiences with injections and implants for contraception; in addition, women had higher stigma scores than men (2.27 vs. 2.13, *p* = 0.04), which may also have impacted preferences. Another possibility is that medical mistrust is more prevalent in the Atlanta participant sub‐population, which was more likely to be Black. Of note, in an extension of the ATLAS study that investigated the safety of “direct‐to‐injection” cabotegravir/rilpvirine initiation as an option alongside the standard pre‐treatment negative reaction testing, Black participants were less likely than White participants to opt for “direct to injection” [40]. Future studies of patient preferences for LA‐ART options should consider including measurement of medical mistrust and evaluating structural barriers to treatment access that may alter preferences.

This study has a number of limitations. First, we only targeted patients who were engaged in HIV care at our clinical sites, and so missed PWH in the study areas who were not diagnosed or engaged in care. In addition, patients who volunteered for the study, especially those who participated online, may differ from those who did not. Second, our work was conducted in only two U.S. regions and our study population is, therefore, not representative of PWH in other geographic areas or settings. While an online study could potentially reach a larger group of PWH, there is a trade‐off between the challenges of online recruitment and recruitment from clinical settings in which HIV status can be confirmed and other clinical data are available. Third, we may have excluded potential LA‐ART products (e.g. infusions of broadly neutralizing antibodies) that may come on the market eventually or included product modalities that may turn out not to be feasible. That said, we developed our DCE based on feedback from 12 experts in the field in the year immediately prior to study launch, using the best information available at the time. Fourth, we were unable to fully examine differences between subcutaneous and intramuscular injections due to the complexities of the restrictions used and did not compare regimens that included mixed modalities due to concerns about cognitive overload. In addition, because the home location was restricted to those modes that could be self‐administered with ease, we could not distinguish preference for home location from preference for self‐administration. Fifth, DCEs ask individuals to make hypothetical choices, which may differ from choices patients would actually make when opportunities arise in the real world, especially if recommended by their physician. Finally, our focus was to provide a comprehensive assessment of preferences within the study sample, and additional exploration is needed in order to assess preference heterogeneity across participant sub‐groups; this work will be forthcoming. Despite these limitations, the present analysis builds on prior patient preference research on LA‐ART by expanding the options participants considered beyond the injectable products first on the market, and is, therefore, a unique and valuable contribution.

## 5 CONCLUSIONS

In conclusion, PWH in the United States may soon have several options for LA‐ART. Our results suggest that LA oral tablets will be preferred by many patients over their current treatment, while implants and injections with longer duration may be acceptable to some patients. Future research should investigate sources of preference heterogeneity and actual uptake of and retention on products, when available.

## COMPETING INTERESTS

BH is an employee of Pfizer. SMG has received support from Gilead and Cepheid for her research. VCM has received investigator‐initiated research grants (to the institution) and consultation fees (both unrelated to the current work) from Eli Lilly, Bayer, Gilead Sciences and ViiV. The other authors declare that they have no competing interests directly relevant to the content of this article.

## AUTHORS’ CONTRIBUTIONS

SMG, JMS, BH and DB designed the study; SMG and JMS acquired funding; VCM oversaw the Atlanta site; ATB and the Emory PREFER Team collected data; DB and ES analysed the data; ACC and RJYH contributed expertise in long‐acting treatment development and testing; and SMG wrote the initial manuscript. All authors contributed to and approved the final manuscript.

## FUNDING

Financial support for this study was provided by National Institutes of Health (NIH) grant R01 MH121424, the University of Washington/Fred Hutch Center for AIDS Research (P30 AI027757) and the Emory University Center for AIDS Research (P30AI050409). SMG and JMS were also supported by the University of Washington Behavioral Research Center for HIV (BIRCH), an NIMH‐funded programme (P30 MH123248). RJYH and ACC are funded in part by NIH grants UM1 AI120176; AI 148055; AI149665; and UNITAID 2020‐39‐GLAD. The funding agreements ensured the authors’ independence in designing the study, interpreting the data, and writing and publishing the report. This project also utilized REDCap electronic data capture, which is supported at the University of Washington by grants UL1 TR002319, KL2 TR002317 and TL1 TR002318 from NCATS/NIH.

## Supporting information


**Supporting Information 1**. Project PREFER SurveyEngine Questionnaire & DCE

**Figure S1A**. Long‐acting antiretroviral treatment (LA‐ART) preference weights from conditional logistic regression for Seattle participants.

**Figure S1B**. Long‐acting antiretroviral treatment (LA‐ART) preference weights from conditional logistic regression for Atlanta participants.

**Table S1**. Point estimates and 95% confidence intervals from logistic regression for preference weights, Seattle participants.
**Table S2**. Point estimates and 95% confidence intervals (CI) from conditional logistic regression for preference weights, Atlanta participants.

## Data Availability

De‐identified data will be made available in the Harvard Dataverse upon publication.
