# Supplementary material for: U.S. patient preferences for long‐acting HIV treatment: a discrete choice experiment
Source: J Int AIDS Soc. 2023 Jul 13;26(Suppl 2):e26099. doi: 10.1002/jia2.26099 (PMC10338996; doi:10.1002/jia2.26099)
Supplement: Supplementary file 1 — Supporting Information 1. Project PREFER SurveyEngine Questionnaire & DCE [file JIA2-26-e26099-s001.docx]

Study ID#: [program as XXXNNN]

Date: [program as MM/DD/YYYY]

Location of Interview:

Evaluator initials: _______________

**Introduction**

This study is about HIV treatment options.

- Taking HIV treatment regularly allows many people living with HIV to have undetectable viral loads.
- Having an undetectable viral load means your health is better and you have no risk of spreading HIV to others.
- But some people have a hard time taking HIV medication every day.

New HIV treatment options in development will not need to be taken every day – they are long-acting.

- They may be given as pills or in other ways, such as injections or implants.
- These new options may make it easier for people to take HIV treatment.

In this study, we are asking people living with HIV how they feel about new options for long-acting treatment.

- We would like to know if the new options are acceptable and how they compare to daily pills.

We will ask you a series of questions:

- First, we will ask about your current HIV treatment regimen.
- Then, we will show you a series of hypothetical new treatment options and ask which you prefer, compared to your current HIV regimen.

By completing this study, you will help us identify the options most acceptable to people living with HIV.

**Treatment Options**

We will describe the four ways that long-acting treatment can be taken, so that you understand how the treatments differ from one another.

Treatment option 1. Injections under the skin:

- These injections would be similar to insulin injections for diabetes.
- They use a short, thin needle, and are usually not very painful. They may cause slight bruising at the injection site.
- For this type of injection, the skin on the belly or thigh is lightly pinched or “bunched” together. Then the needle is inserted under the skin at a 45-degree angle before the plunger is used to push the medication out of the syringe.
- Most regimens for HIV treatment will include two injections each time you receive a dose.


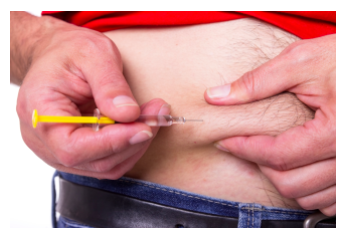


Treatment option 2. Injections into the muscle:

- These injections are typically given in the buttocks or upper arm, similar to a vaccination.
- Injections into muscle tend to be slightly more painful and leave more bruising at the injection site than injections under the skin.
- Most regimens for HIV treatment will include two injections each time you receive a dose, one in each buttock or arm.


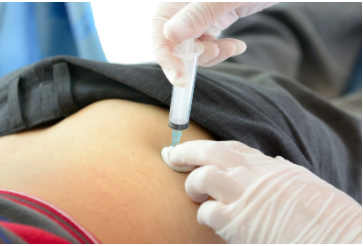


Treatment option 3. Implants:

- Implants are usually inserted in the arm near the bicep, after an injection of local anesthetic (numbing agent) to numb the area before insertion.
- Insertion can cause some pain and significant bruising, and can cause a small scar to form where the implants were inserted.
- Once inserted, the implants release the medication slowly until all the medication is used up.
- If there are side effects or other problems due to the implant, it can be removed right away so that the medication is no longer in the body.
- Once the medication is used up, the implant needs to be removed and replaced.
- The outline of the implant can be seen under your skin, as shown in the photo below.
- Most regimens for HIV treatment will include two implants each time you get them, one inserted right next to the other.


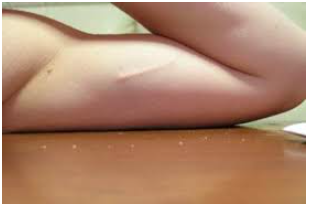


Treatment Option 4. Long-acting oral pills:

- These are pills people swallow, similar to what most people currently take to treat HIV.
- The effects of long-acting pills last longer so they do not have to be taken every day.
- Most long-acting oral regimens for HIV treatment will be given in one or two pills each time you take them.


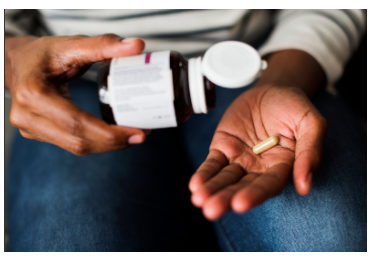


*This is a lot of information, so let’s stop to review and make sure you understand.*

Based on the descriptions of the treatment options, please indicate whether the following statements are true or false:

CQ1. An injection under the skin tends to be more painful than an injection into the muscle.

- 1. If “True” selected:
     - Sorry, that is incorrect. Remember, an injection under the skin is less painful usually than an injection under the muscle. Please click "next" to try this question again.
  2. If “False” selected:
     - Great job! That is correct.

CQ2. The outline of an implant can be seen under the skin.

1. If “True” selected:
   - Great job! That is correct.
2. If “False” selected:

- Sorry, that is incorrect. Remember, the outline of an implant can be seen under the skin.

CQ3. Currently, most patients take one or more tablets by mouth each day for HIV treatment.

1. If “True” selected:
   - Great job! That is correct.
2. If “False” selected:
   - Sorry, that is incorrect. Most patients do currently take one or more tablets by mouth each day for their HIV treatment. Please click "next" to try this question again.

**Features**

The four options for new treatments (injections under the skin, injections into muscle, implants, and long-acting pills) differ from each other in important ways. We describe these differences on the next few screens.

Feature 1

*Location of treatment: where would I get this treatment?*

For example, an oral medication that you take at home has home as its location of treatment.

The new treatment options may have the following options for location:

- Home
- Local pharmacy
- Clinic or doctor's office

If the location is home, the treatment would be self-administered. Treatments given at the local pharmacy, clinic, or doctor’s office would be administered by a trained professional.

Feature 2

*Frequency of dosing: how often would I get this treatment?*

For example, a daily medication is taken every day.

The new options last longer than daily medication and may be taken:

- Once a week
- Once a month
- Once every 2 months
- Once every 3 months
- Once every 6 months
- Once a year

Feature 3

*Pain: how much pain would I feel?*

For example, an oral medication does not cause any pain.

The new treatment options may cause the following levels of pain:

- None
- Mild
- Moderate

**Assumptions**

We are now going to ask you to think about different treatment options. When you do, please assume the following:

1. First, please assume all of the treatment options ***work equally well***, meaning that all options can lead to undetectable viral load. Just like the oral pills, none of the options we show you will cure your HIV infection.

2. Second, please assume there would be ***no difference in cost*** to you compared to what you pay for your current HIV treatment.

3. Third, please assume that the safety of these treatment location options (e.g., clinic, pharmacy) will ***not be affected by COVID-19***.

**Now, we will show you a 1 minute video with an example of a choice you will be asked to make, between 2 hypothetical treatments and your current HIV treatment regimen.**

**Please make sure your volume is turned up. If you put the video into full screen mode, you can use the escape key to exit full screen mode.**

**After the video is complete, please minimize the video and click the "next" button in the bottom-right. You will be allowed to click and choose one of the 3 options on the next page.**


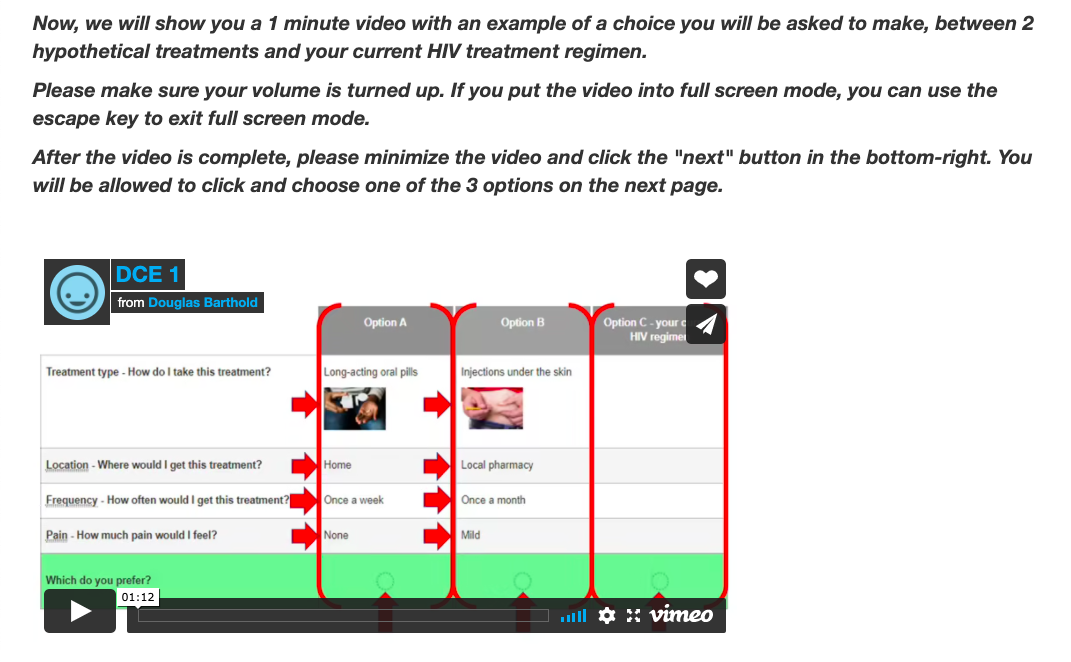


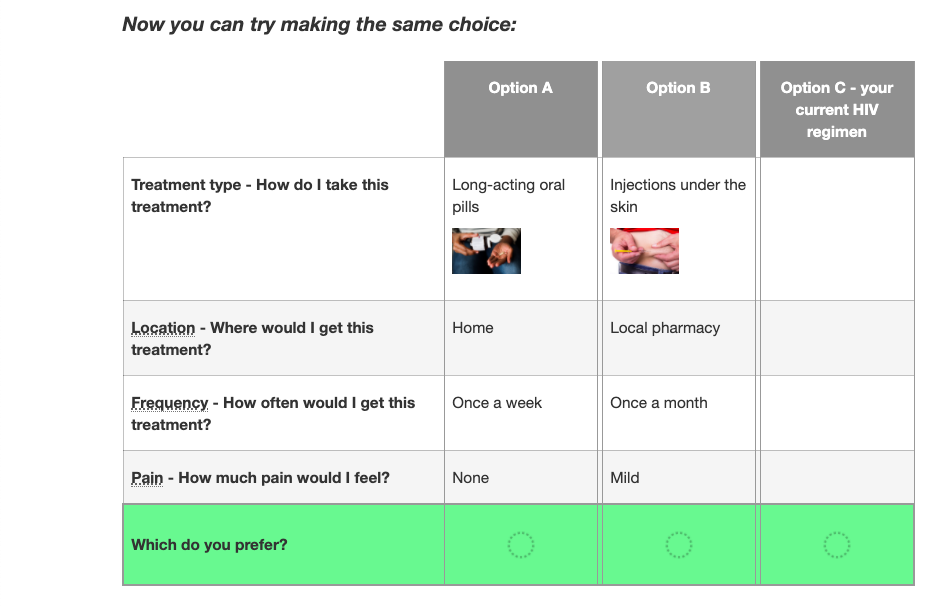


Feature 4

*Pre-treatment undetectability: how long would I need to be undetectable on daily pills before starting this treatment?*

Some long-acting treatments may not be powerful enough to reduce HIV viral load when it is very high. Before starting such treatments, it may be necessary to already have undetectable viral load test results obtained 3 or 6 months before you can switch to the long-acting treatment.

Other long-acting treatments may be powerful enough to make your viral load undetectable even if it is very high when you start.

The options for the amount of time you would need to be undetectable before you can start the long-acting treatment are:

- None
- 3 months
- 6 months

OK, now we will describe a few other important features of the treatment options.

Feature 5

*Pre-treatment negative reaction testing: would I need to take daily pills to check for negative reactions before starting this treatment?*

Some long-acting treatments can cause negative reactions in some people, such as an allergic rash or abnormal liver test results. This is rare, but it can happen. For these treatments, you may need to take the daily oral version of the same medication for approximately 4 weeks to make sure you do not have negative reactions.

If you do have negative reactions, the oral treatment would be stopped immediately and you would not be started on the long-acting version.

Other long-acting treatments may have such a low risk for negative reactions, that they can be started right away.

The options for needing to take an oral treatment for 4-weeks to check for negative reactions before you can start the long-acting treatment are:

- Not needed
- Needed

**OK, we'll now ask you a few questions to make sure this is all clear.**

CQ4. I may need to take daily oral treatment for a time to make sure I do not have negative reactions to the treatment, or so that I can become undetectable before starting the long-acting treatment

1. If “True” selected:
   - Great job! That is correct.
2. If “False” selected:
   - Sorry, that is incorrect. Daily oral treatment may be needed for a time in order to make sure you do not have negative reactions to the treatment or to become undetectable before switching to the new, long-acting treatment. Please click "next" to try this question again.

Feature 6

*Late dose leeway: how late can I be for a dose of this treatment and still remain undetectable?*

Keeping your viral load undetectable requires that you take your treatment as often as it is prescribed.

For most treatments, you can be a little late taking your dose and still be OK.

For example, if you are using a treatment that is usually given every 6 months, you might have up to 3 months after a missed dose as a “buffer” before your HIV viral load becomes detectable and your risk for drug resistance increases.

The same treatment with only 1 month of leeway after a missed dose would have a shorter late dose leeway.

The options for the late dose leeway are:

- Shorter (for example, 1 month)
- Longer (for example, 3 months)

*This is a lot of information, so let's pause and make sure you understand the concept behind "late dose leeway".*

CQ5. The "late dose leeway" is how late you can be for your next dose before your viral load becomes detectable and the risk for drug resistance increases.

- 1. True
     - comp5end: Great job! That is correct.
  2. False
     - comp5incorrect: Sorry, that is incorrect. “Late dose leeway” is a term used to describe how late you can be for your next dose without increasing your risk for drug resistance. Please click "next" to try this question again.
     - Comp5.2: The "late dose leeway" is how late you can be for your next dose before your viral load becomes detectable and the risk for drug resistance increases.
       - TRUE
         - comp5end: Great job! That is correct.
       - FALSE
         - comp5end: Sorry, that is incorrect. “Late dose leeway” is a term used to describe how late you can be for your next dose without increasing your risk for drug resistance.

**Here's another 1 minute video with an example of a choice you will be asked to make, between 2 hypothetical treatments and your current HIV regimen.**

**Please make sure your volume is turned up. If you put the video into full screen mode, you can use the escape key to exit full screen mode.**

**After the video is complete, please minimize the video and click the "next" button in the bottom-right. You will be allowed to click and choose one of the 3 options on the next page.**


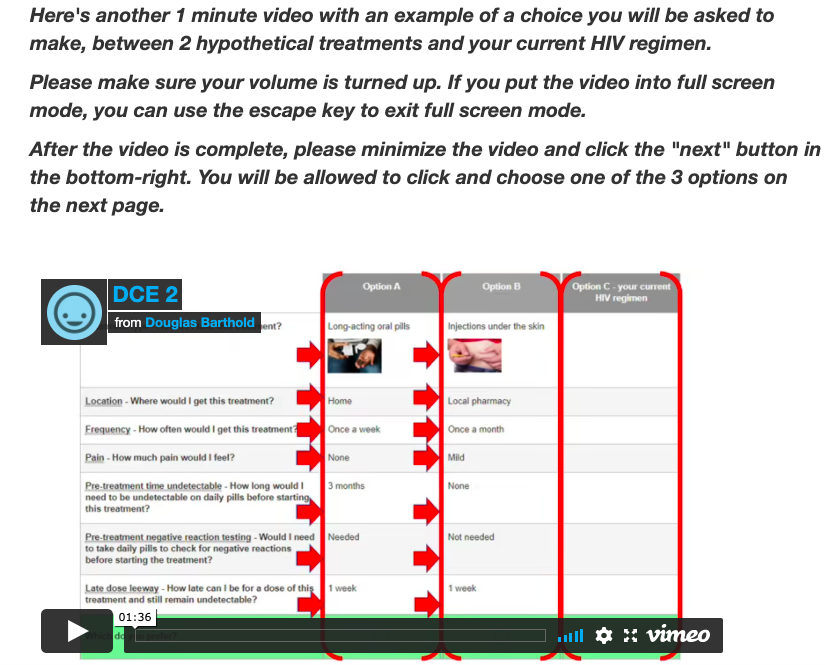


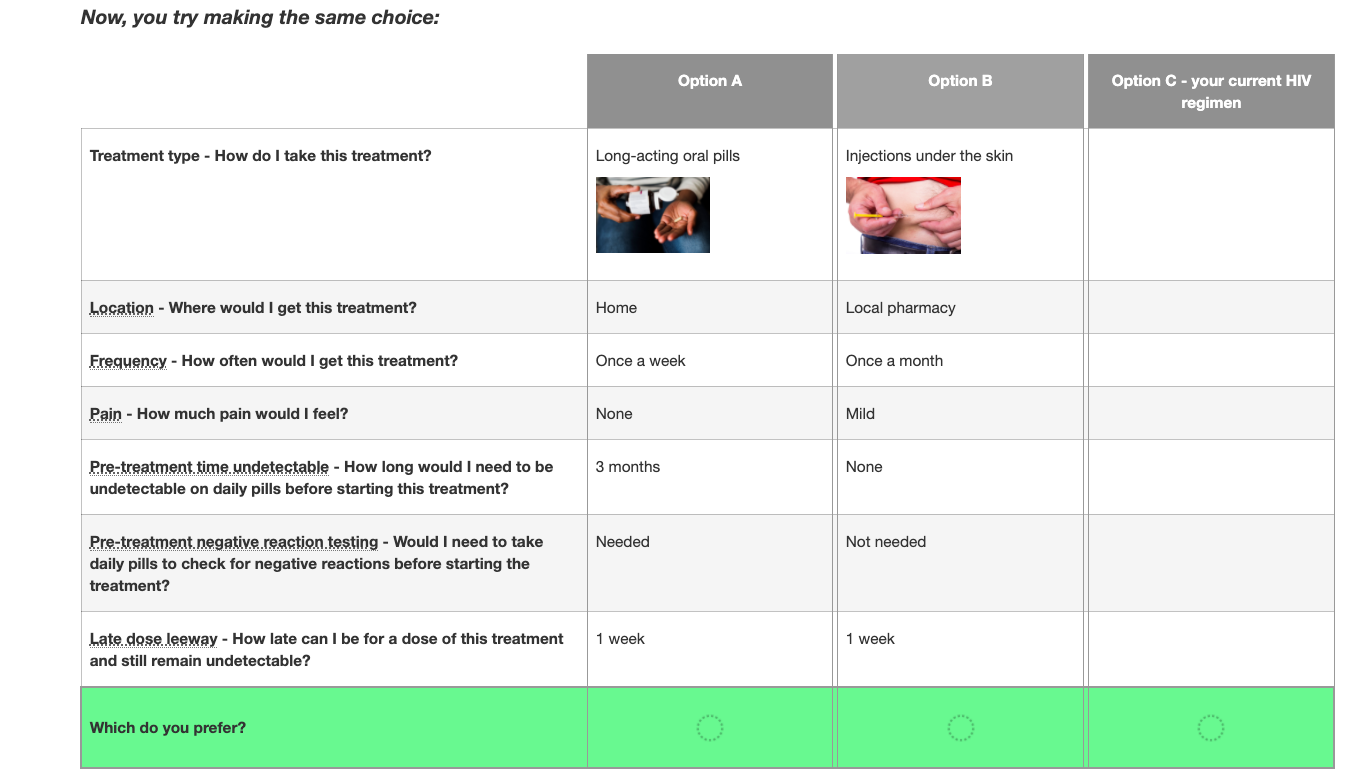


*Great job! You have completed the introduction to the study. Try to keep what you have learned here in mind as you go through the survey.*

**HIV HISTORY**

1. In which year did you first receive your HIV diagnosis? If you're not sure, please give your best estimate.

[NNNN]

Prefer not to say

1. (If year of diagnosis >=2020) In what month did you receive your HIV diagnosis? If you're not sure, please give your best estimate.

[month]

Prefer not to say

1. In what year did you start HIV treatment? If you're not sure, please give your best estimate.

*Error text: The year you started HIV ART treatment needs to be later than or equal to the year you were diagnosed with HIV.*

[NNNN]

Prefer not to say

1. (If year of initiation ≥2020) In what month did you start HIV treatment? If you're not sure, please give your best estimate.

*Error text: The month you started HIV ART has to be equal to or earlier than the month you were diagnosed with HIV.*

[month]

Prefer not to say

1. Have you ever received an AIDS diagnosis? This would be a result of a CD4 count <200 or an opportunistic infection due to low immunity.

a) No

b) Yes

c) Prefer not to say

1. (If year of initiation ≥2022) Have you ever had an undetectable viral load?
2. No
3. Yes
4. Not sure
5. Prefer not to say
6. (If ever detectable, and initiation before 2022) Has your viral load been undetectable for the last 6 months?
   1. No
   2. Yes
   3. Not sure
   4. Prefer not to say
7. (If initiation before 2022) In the last 30 days, on how many days did you miss at least one dose of any of your HIV medications?
   1. [NN] Days [range check 0 through 30],
   2. Don’t know/Don’t remember
   3. Prefer not to say
8. (If initiation before 2022) In the last 30 days, how often did you take your HIV medications in the way you were supposed to?
9. Never
10. Rarely
11. Sometimes
12. Usually
13. Almost always
14. Always
15. Prefer not to say
16. (If initiation before 2022) In the last 30 days, how good a job did you do at taking your HIV medication in the way you were supposed to?
17. Very poor
18. Poor
19. Fair
20. Good
21. Very good
22. Excellent
23. Prefer not to say

**CURRENT AND PAST MEDICATION REGIMENS**

1. (If initiation before 2022) How many ***HIV pills total*** are you prescribed to take each day?
2. 1
3. 2
4. 3+
5. Prefer not to say
6. How many pills are you prescribed to take each day to treat something ***other than HIV*** infection? Enter number of pills prescribed by your doctor, NOT counting HIV pills and NOT counting over-the-counter medications, vitamins, and supplements.

Please enter only a number between 0 and 20 (1, 2, 3, etc.)

[NN, with range check 0 to 20]

Don’t know/Don’t remember

Prefer not to say

1. (If initiation before 2022) How many different ***HIV treatment regimens*** (i.e., combinations of drugs) have you ever been prescribed? Please include your current one.

a) none

b) 1

c) 2

d) 3+

e) Prefer not to say

**EXPERIENCE WITH INJECTIONS**

1. How often have you ever given yourself an injection for medical treatment or recreational drug use?
2. Never
3. A few times
4. Many times
5. Prefer not to say
6. How often has someone else given you an injection for medical treatment or recreational drug use?
7. Never
8. A few times
9. Many times
10. Prefer not to say
11. How much do you agree or disagree with this statement?

*I HATE getting injections and try to avoid getting them whenever possible.*

1. Strongly agree
2. Somewhat agree
3. Neither agree nor disagree
4. Somewhat disagree
5. Strongly disagree
6. Prefer not to say

**PILL STORAGE AND CLINIC VISITS**

1. What is your current living situation?

a) My own house/apartment

b) Someone else's house/apartment

c) Don’t have a stable living situation right now

d) Prefer not to say

1. In your current living situation, do you have a secure place to store your

HIV medication?

a) No

b) Yes

c) Prefer not to say

1. In your current living situation, do you have a refrigerator where you can store medications if needed?

a) No

b) Yes

c) Prefer not to say

1. How often do you spend one night or more away from home?
2. Never
3. Less than once a year
4. At least once a year, but not monthly
5. At least once a month, but not weekly
6. At least once a week
7. Prefer not to say
8. How long does it take for you to get from where you live to the clinic where you receive HIV care?

a) 0 to 15 minutes

b) 15 to 30 minutes

c) 30 minutes to 1 hour

d) Over an hour

e) Prefer not to say

1. How do you get from where you live to the clinic where you receive HIV care?
2. Personal vehicle
3. Friend/Family drives me
4. Public Transportation
5. Lyft/Uber/Taxi
6. Walk/Bicycle
7. Special Transport Services
8. Prefer not to say
9. In general, how easy is it for you to get to the clinic where you receive HIV care and treatment?

a) Very easy

b) Easy

c) Neutral

d) Difficult

e) Very difficult

f) Prefer not to say

1. How much do you agree or disagree with this statement:

Clinic waiting times make it difficult/inconvenient for me to receive my HIV care.

a) Strongly agree

b) Somewhat agree

c) Neither agree nor disagree

d) Somewhat disagree

e) Strongly Disagree

f) Prefer not to say

**DISCRETE CHOICE EXPERIMENT – 17 Choice Sets**

**﻿***In the following questions, you will be asked to choose between 2 hypothetical treatments, and your current therapy.*

*Please assume that your current therapy is a daily oral pill.*

**
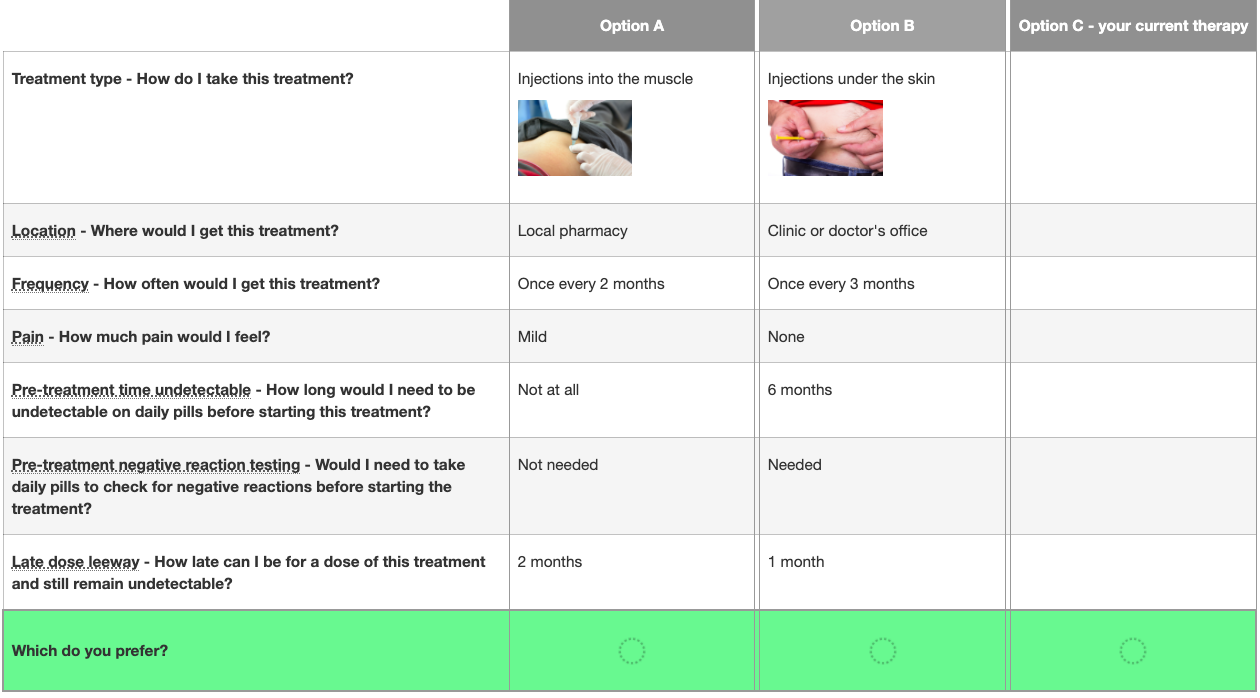
**

*You will now be asked to complete 17 treatment choices. While this may seem repetitive, the options will keep changing, so it is important that you read each alternative carefully.*

*Each choice is between 2 hypothetical treatment options, and your current therapy.*

**CHOICE SETS HERE**

**QUALITY OF LIFE**

1. How would you rate the quality of your life?
2. Very Poor
3. Poor
4. Neither poor nor good
5. Good
6. Very good
7. Prefer not to say
8. How satisfied are you with your health?
9. Very dissatisfied
10. Dissatisfied
11. Neither satisfied nor dissatisfied
12. Satisfied
13. Very Satisfied
14. Prefer not to say

**PROVIDER AND OTHER SUPPORT**

1. Do you have a primary provider that you see for your HIV care?

a) No

b) Yes

c) Prefer not to say

1. (If yes to question 27) How would you rate the strength of your relationship with this provider?

a) Very poor

b) Poor

c) Fair

d) Good

e) Very good

f) Excellent

g) Prefer not to say

**HIV Stigma Scale**

This section asks about some of the social and emotional aspects of having HIV. Some of these issues may be sensitive. Please do your best to respond to each statement.

How much do you disagree or agree with the following statements?

1. It is difficult to tell people about my HIV infection.
   1. Strongly agree
   2. Agree
   3. Disagree
   4. Strongly Disagree
   5. Prefer not to say
2. Being HIV positive makes me feel dirty.
   1. Strongly agree
   2. Agree
   3. Disagree
   4. Strongly Disagree
   5. Prefer not to say
3. I feel guilty that I am HIV positive.
4. Strongly agree
5. Agree
6. Disagree
7. Strongly Disagree
8. Prefer not to say
9. I am ashamed that I am HIV positive.
10. Strongly agree
11. Agree
12. Disagree
13. Strongly Disagree
14. Prefer not to say
15. I sometimes feel worthless because I am HIV positive.
16. Strongly agree
17. Agree
18. Disagree
19. Strongly Disagree
20. Prefer not to say
21. I hide my HIV status from others.
22. Strongly agree
23. Agree
24. Disagree
25. Strongly Disagree
26. Prefer not to say

**MULTIDIMENSIONAL SCALE OF PERCEIVED SOCIAL SUPPORT**

We are interested in how you feel about the following statements. Read each statement carefully. Indicate how you feel about each statement.

1. There is a person who is around when I am in need.
2. Strongly disagree
3. Mildly disagree
4. Neutral
5. Mildly agree
6. Strongly agree
7. Prefer not to say
8. There is a special person with whom I can share my joys and sorrows.
9. Strongly disagree
10. Mildly disagree
11. Neutral
12. Mildly agree
13. Strongly agree
14. Prefer not to say
15. My family really tries to help me.
16. Strongly disagree
17. Mildly disagree
18. Neutral
19. Mildly agree
20. Strongly agree
21. Prefer not to say
22. I get the emotional help and support I need from my family.
23. Strongly disagree
24. Mildly disagree
25. Neutral
26. Mildly agree
27. Strongly agree
28. Prefer not to say
29. I have a special person who is a real source of comfort to me.
30. Strongly disagree
31. Mildly disagree
32. Neutral
33. Mildly agree
34. Strongly agree
35. Prefer not to say
36. My friends really try to help me.
37. Strongly disagree
38. Mildly disagree
39. Neutral
40. Mildly agree
41. Strongly agree
42. Prefer not to say
43. I can count on my friends when things go wrong.
44. Strongly disagree
45. Mildly disagree
46. Neutral
47. Mildly agree
48. Strongly agree
49. Prefer not to say
50. I can talk about my problems with my family.
51. Strongly disagree
52. Mildly disagree
53. Neutral
54. Mildly agree
55. Strongly agree
56. Prefer not to say
57. I have friends with whom I can share my joys and sorrows.
58. Strongly disagree
59. Mildly disagree
60. Neutral
61. Mildly agree
62. Strongly agree
63. Prefer not to say
64. There is a special person in my life who cares about my feelings.
65. Strongly disagree
66. Mildly disagree
67. Neutral
68. Mildly agree
69. Strongly agree
70. Prefer not to say
71. My family is willing to help me make decisions.
72. Strongly disagree
73. Mildly disagree
74. Neutral
75. Mildly agree
76. Strongly agree
77. Prefer not to say
78. I can talk about my problems with my friends.
79. Strongly disagree
80. Mildly disagree
81. Neutral
82. Mildly agree
83. Strongly agree
84. Prefer not to say

**SOCIODEMOGRAPHIC FACTORS**

1. Are you of Hispanic/Latino/Chicano origin?
2. No
3. Yes
4. Prefer not to say
5. Which of the following best describes your racial/ethnic background?
6. American Indian or Alaskan Native
7. Asian
8. Native Hawaiian or other Pacific Islander
9. Black or African American
10. White
11. None of the above
12. Prefer not to say
13. Other
14. What is the language in which you read and write best?
15. English
16. Other language
17. Prefer not to say
18. What is your gender identity?
19. Male
20. Female
21. Trans Man/Female to Male (FTM)
22. Transgender Female/ Trans Women/Male to Female (MTF)
23. Prefer not to say
24. Other (please specify)
25. What sex were you assigned at birth on your original birth certificate?
26. Male
27. Female
28. Prefer not to say
29. Other (please specify)
30. Do you think of yourself as…
31. Straight or heterosexual
32. Gay or lesbian
33. Bisexual
34. Prefer not to say
35. Other (please specify)
36. Which of the following best describes your religious background?
37. Atheist or agnostic
38. Buddhist
39. Catholic
40. Christian, non denominational
41. Hindu
42. Jehovah's Witness
43. Jewish
44. Muslim
45. Protestant
46. Seventh Day Adventist
47. Sikh
48. No affiliation
49. Prefer not to say
50. Other (please specify)
51. Do you have a steady sexual/romantic partner?
52. No
53. Yes
54. Prefer not to say
55. (If yes to Question 54) Do you live with this person?
56. No
57. Yes
58. Prefer not to say
59. Which of the following best describes your current occupational status? Please check all that apply.
60. Paid work full-time
61. Paid work part-time
62. Unemployed
63. Full-time student
64. Not working due to disability
65. Retired
66. Prefer not to say
67. What is the highest level of education you have completed?
68. Less than high school
69. High school graduate or GED
70. Some college/AA degree/Technical school training
71. College graduate (BA or BS)
72. Graduate degree: Master’s or Doctorate degree (MD, PhD, JD)
73. Prefer not to say
74. Approximately how much income do you receive each month from all sources including employment, public assistance, and other activities?

a) $0-1000

b) $1001-2000

c) $2001-3000

d) Greater than $3000

e) Prefer not to say

1. Do you currently have health insurance?
2. No
3. Yes
4. Prefer not to say

1. (If yes to question 59) What type of insurance do you have?
2. Private insurance through employer
3. Private insurance through spouse’s employer
4. Private insurance purchased through health exchange
5. Private insurance through my parents
6. Public insurance such as: Medicare, Medicaid, Apple Health, VA
7. No insurance
8. Other
9. Prefer not to say
10. (if started ART before 2022) How much do you pay for each refill of your current HIV medications per month?

a) $0

a) $1-$10

b) $11-$25

c) $26-$50

d) More than $50

e) Prefer not to say

**REMINDER STRATEGIES**

1. (If started ART before 2022) Do you currently use any of the following strategies as reminders to take your daily HIV pills? Check all that apply.
   1. Partner, friend, or family reminds me in person
   2. Partner, friend, or family reminds me by text message/SMS
   3. Alarm on cell phone or other alarm
   4. App on phone
   5. Clinic sends text message/SMS
   6. Clinic sends e-mail or eCare message
   7. Video call with clinic
   8. Pillbox or mediset
   9. None of these
   10. Prefer not to say
   11. Other (please specify)
2. (If app on phone=yes) Which app do you use on your phone?

[free form text]

1. If you were to switch to a long-acting HIV treatment that is not taken daily, would you want to use any of the following strategies as reminders of when your next dose is due? Check all that apply.
   1. Partner, friend, or family reminds me in person
   2. Partner, friend, or family reminds me by text message/SMS
   3. Alarm on cell phone or other alarm
   4. App on phone
   5. Clinic sends text message/SMS
   6. Clinic sends e-mail or eCare message
   7. Video call with clinic
   8. Prefer not to say
   9. Other (please specify)
2. When choosing between hypothetical treatments, did you find yourself making choices based on one or two of the features of the treatment options?
3. Yes, I based choices on one or two features
4. No, I considered ALL features
5. Prefer not to say
6. (If based on one or two features) If you based your choices on just one or two features, why didn't you consider ALL the features?
7. There were just too many features to include in my decisions
8. The other features were too complicated or it was unclear what they meant
9. Prefer not to say
10. Other reason (please explain)
11. Prefer not to say

**COVID-19**

We would like to understand the impact of COVID-19 on your life. Please state your level of agreement with the following statements.﻿

1. COVID-19 impacted my day-to-day life.
2. I strongly disagree
3. I somewhat disagree
4. Neutral
5. I somewhat agree
6. I strongly agree
7. Prefer not to say
8. I am afraid of getting COVID-19.
9. I strongly disagree
10. I somewhat disagree
11. Neutral
12. I somewhat agree
13. I strongly agree
14. Prefer not to say

**Submit and Finish**

Please click the button below to complete the survey.

You will receive your gift card within 3 business days.

Thank you!
