# Supplementary figures and images for: U.S. patient preferences for long‐acting HIV treatment: a discrete choice experiment
Source: J Int AIDS Soc. 2023 Jul 13;26(Suppl 2):e26099. doi: 10.1002/jia2.26099 (PMC10338996; doi:10.1002/jia2.26099)

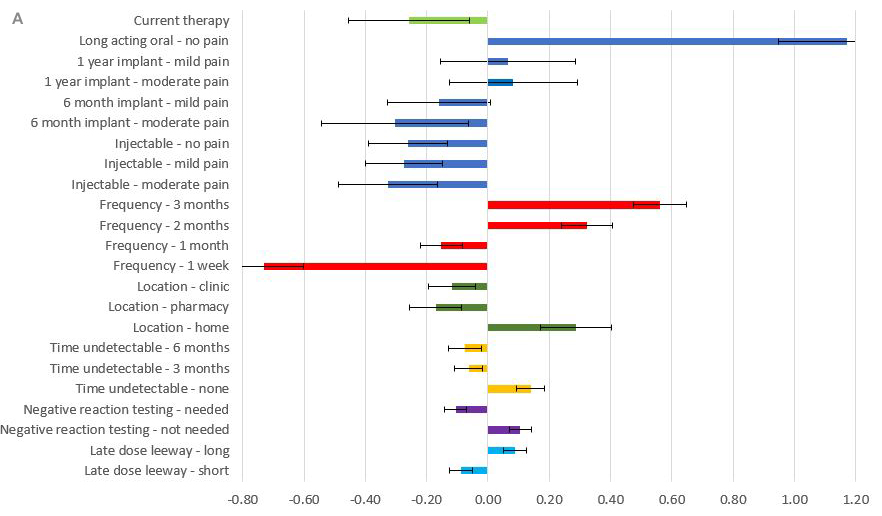

Supplement: Supplementary file 2 — Figure S1A. Long‐acting antiretroviral treatment (LA‐ART) preference weights from conditional logistic regression for Seattle participants. [file JIA2-26-e26099-s004.JPG]

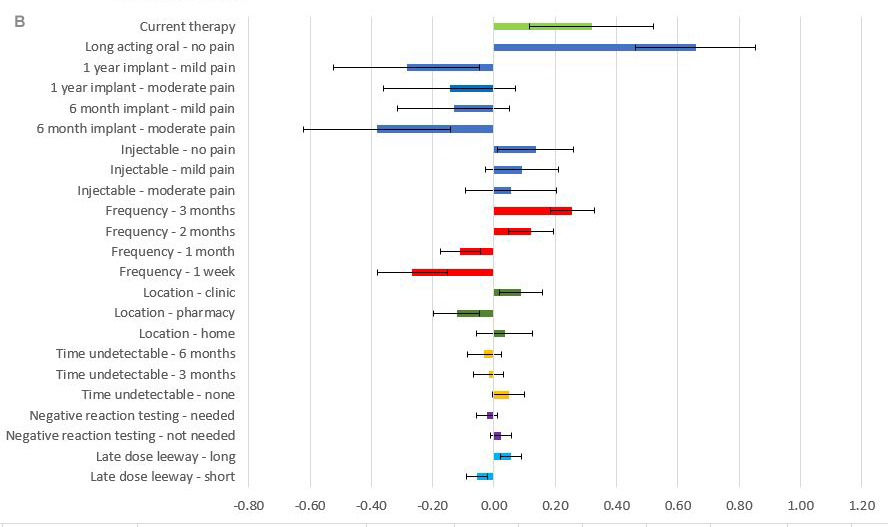

Supplement: Supplementary file 3 — Figure S1B. Long‐acting antiretroviral treatment (LA‐ART) preference weights from conditional logistic regression for Atlanta participants. [file JIA2-26-e26099-s003.JPG]
