# Supplementary material for: U.S. patient preferences for long‐acting HIV treatment: a discrete choice experiment
Source: J Int AIDS Soc. 2023 Jul 13;26(Suppl 2):e26099. doi: 10.1002/jia2.26099 (PMC10338996; doi:10.1002/jia2.26099)
Supplement: Supplementary file 4 — Table S1. Point estimates and 95% confidence intervals from logistic regression for preference weights, Seattle participants. Table S2. Point estimates and 95% confidence intervals (CI) from conditional logistic regression for preference weights, Atlanta participants. [file JIA2-26-e26099-s002.docx]

**Supplemental Table 1. Point estimates and 95% confidence intervals from logistic regression for preference weights, Seattle participants.** Thin lines separate the different attributes assessed and an asterisk indicates the omitted level in the regression. Preference weights are relative to the mean effect. Non-overlapping 95% confidence intervals for preference weights of different levels of the same attribute indicate significantly different utilities between the two levels compared.

|  | Preference weight | 95% CI |  |
| --- | --- | --- | --- |
| Current therapy (alternative-specific constant) | -0.26 | -0.46, -0.06 |  |
| Long acting oral - no pain* | 1.17 | 0.95, 1.40 |  |
| 1 year implant - mild pain | 0.07 | -0.16, 0.29 |  |
| 1 year implant - moderate pain | 0.08 | -0.13, 0.29 |  |
| 6 month implant - mild pain | -0.16 | -0.33, 0.01 |  |
| 6 month implant - moderate pain | -0.30 | -0.54, -0.06 |  |
| Injectable - no pain | -0.26 | -0.39, -0.13 |  |
| Injectable - mild pain | -0.27 | -0.40, -0.15 |  |
| Injectable - moderate pain | -0.33 | -0.49, -0.16 |  |
| Frequency - 3 months | 0.56 | 0.48, 0.65 |  |
| Frequency - 2 months | 0.32 | 0.24, 0.41 |  |
| Frequency - 1 month | -0.15 | -0.22, -0.08 |  |
| Frequency - 1 week* | -0.73 | -0.86, -0.60 |  |
| Location - clinic | -0.12 | -0.19, -0.04 |  |
| Location - pharmacy | -0.17 | -0.26, -0.09 |  |
| Location - home* | 0.29 | 0.17, 0.40 |  |
| Time undetectable - 6 months | -0.08 | -0.13, -0.02 |  |
| Time undetectable - 3 months | -0.06 | -0.11, -0.02 |  |
| Time undetectable - none* | 0.14 | 0.09, 0.18 |  |
| Negative reaction testing - needed | -0.11 | -0.14, -0.07 |  |
| Negative reaction testing - not needed* | 0.11 | 0.07, 0.14 |  |
| Late dose leeway – long^a^ | 0.09 | 0.05, 0.13 |  |
| Late dose leeway - short*^a^ | -0.09 | -0.13, -0.05 |  |

^a^ Late dose leeway was defined as the flexibility or “forgiveness” in dosing timing before breakthrough viremia, with long leeway defined as 100% of the dosing interval and “short” leeway defined as 50% of the dosing interval for that specific treatment option.

**Supplemental Table 2. Point estimates and 95% confidence intervals (CI) from conditional logistic regression for preference weights, Atlanta participants.** Thin lines separate the different attributes assessed and an asterisk indicates the omitted level in the regression. Preference weights are relative to the mean effect. Non-overlapping 95% confidence intervals for preference weights of different levels of the same attribute indicate significantly different utilities between the two levels compared.

|  | Preference weight | 95% CI |  |
| --- | --- | --- | --- |
| Current therapy (alternative-specific constant) | 0.32 | 0.12, 0.52 |  |
| Long acting oral - no pain* | 0.66 | 0.46, 0.85 |  |
| 1 year implant - mild pain | -0.28 | -0.52, -0.05 |  |
| 1 year implant - moderate pain | -0.14 | -0.36, 0.07 |  |
| 6 month implant - mild pain | -0.13 | -0.31, 0.05 |  |
| 6 month implant - moderate pain | -0.38 | -0.62, -0.14 |  |
| Injectable - no pain | 0.14 | 0.01, 0.26 |  |
| Injectable - mild pain | 0.09 | -0.03, 0.21 |  |
| Injectable - moderate pain | 0.06 | -0.09, 0.20 |  |
| Frequency - 3 months | 0.26 | 0.18, 0.33 |  |
| Frequency - 2 months | 0.12 | 0.05, 0.19 |  |
| Frequency - 1 month | -0.11 | -0.17, -0.05 |  |
| Frequency - 1 week* | -0.27 | -0.38, -0.15 |  |
| Location - clinic | 0.09 | 0.02, 0.16 |  |
| Location - pharmacy | -0.12 | -0.20, -0.05 |  |
| Location - home* | 0.03 | -0.06, 0.13 |  |
| Time undetectable - 6 months | -0.03 | -0.09, 0.02 |  |
| Time undetectable - 3 months | -0.02 | -0.07, 0.03 |  |
| Time undetectable - none* | 0.05 | 0.00, 0.10 |  |
| Negative reaction testing - needed | -0.02 | -0.06, 0.01 |  |
| Negative reaction testing - not needed* | 0.02 | -0.01, 0.06 |  |
| Late dose leeway - long^a^ | 0.06 | 0.02, 0.09 |  |
| Late dose leeway - short*^a^ | -0.06 | -0.09, -0.02 |  |

^a^ Late dose leeway was defined as the flexibility or “forgiveness” in dosing timing before breakthrough viremia, with long leeway defined as 100% of the dosing interval and “short” leeway defined as 50% of the dosing interval for that specific treatment option.
